# A Polarization-Dependent Normal Incident Quantum Cascade Detector Enhanced Via Metamaterial Resonators

**DOI:** 10.1186/s11671-016-1749-2

**Published:** 2016-12-01

**Authors:** Lei Wang, Shen-Qiang Zhai, Feng-Jiao Wang, Jun-Qi Liu, Shu-Man Liu, Ning Zhuo, Chuan-Jin Zhang, Li-Jun Wang, Feng-Qi Liu, Zhan-Guo Wang

**Affiliations:** Key Laboratory of Semiconductor Materials Science, Institute of Semiconductors, Chinese Academy of Sciences, University of Chinese Academy of Sciences; Beijing Key Laboratory of Low Dimensional Semiconductor Materials and Devices, P.O. Box 912, Beijing, 100083 People’s Republic of China

**Keywords:** Infrared, Photodetectors, Polarization-selective devices, Metamaterials

## Abstract

The design, fabrication, and characterization of a polarization-dependent normal incident quantum cascade detector coupled via complementary split-ring metamaterial resonators in the infrared regime are presented. The metamaterial structure is designed through three-dimensional finite-difference time-domain method and fabricated on the top metal contact, which forms a double-metal waveguide together with the metallic ground plane. With normal incidence, significant enhancements of photocurrent response are obtained at the metamaterial resonances compared with the 45° polished edge coupling device. The photocurrent response enhancements exhibit clearly polarization dependence, and the largest response enhancement factor of 165% is gained for the incident light polarized parallel to the split-ring gap.

## Background

High-performance infrared photodetectors operating in the range of 3–14 μm are highly needed for a variety of applications, such as security surveillance, chemical sensing, and industrial process monitoring [1]. This has promoted increasing research interest in new materials and structures to improve detector performances, such as spectral sensitivity, leakage current level, and operating temperature. Thereinto, semi-conductor intersubband photodetectors have been widely explored due to their capabilities of flexible energy-band tailoring and ultrahigh-speed operation [2, 3]. Inspired by the prototype of quantum cascade laser, a new type of semi-conductor intersubband detectors, quantum cascade detectors (QCDs) have been proposed and gained great progresses over the past decade [4]. Based on bound-to-bound intersubband transitions in a built-in asymmetric conduction band structure, QCDs share the advantages of operation wavelength designability, low noise photovoltaic operation mode and therefore room temperature operation capability over well-established detector types [5]. Thus, QCDs are largely free from the limit of the integration time due to capacitance saturation of the read-out circuit and very promising for thermal imaging. Moreover, the thermal load is strongly reduced which is of interest if the available cooling is limited, for example in spaceborne systems.[6] Up to the present, QCDs have covered a large wavelength range from the near-infrared to the terahertz region [7–14]. Many new structure designs have been presented to improved device performances, such as mini-band [12] and energy mini-steps [13] transport designs for very long wave detection and diagonal transition design [15, 16] for an improved overall performance. In addition, QCDs have prove their high-temperature operation capability [9, 17, 18], and exhibited their potential to be used for arrays in imaging applications [19].

However, QCDs suffer from the disadvantage of insensibility to normal incident light and low quantum efficiency, due to their intersubband transition principle [20]. Thus, many novel coupled structures have been applied to fulfill the intersubband transition selection rule and enhance quantum efficiency [21, 22]. Metamaterials are man-made structures compositing of sub-wavelength periodically arranged metallic resonators that allow for three-dimensional control of light [23]. Utilization of resonant inclusions allows for the realization of stunning optical properties that cannot be found otherwise in nature, such as superlensing, negative refraction, or cloaking. These exotic properties strongly depend on the geometry of metamaterial molecules rather than their composition. Since they were theoretically proposed by Pendry et al. [24] and experimentally demonstrated by Smith et al. [25], metamaterials have attracted intensive research interest and extended to infrared region [26, 27] in recent years because of their wide applications in super lenses, slow light, data storage, optical switching, and so on. Another property of interest is that metamaterial resonators can excite a strong field enhancement in the near-field and be used as highly sub-wavelength electromagnetic cavities [28]. This property makes them the ideal candidates for coupling incoming light to a semi-conductor medium and thus a promising method to enhance absorption in semi-conductor intersubband photodetectors. A. Benz et al. have innovatively applied a complementary split-ring resonator (CSRR) on a quantum-cascade-laser structure to enhanced photovoltaic detection in terahertz region and brought in inspiring results [29]. In this work, a similar structure is integrated on a quantum cascade detector in infrared region to enhance device response and, in further, the polarization selective enhancement property of the integrated device has been focused on. The metamaterial structure is designed through three-dimensional finite-difference time-domain (FDTD) method and directly fabricated into the top metal contact, which forms a double-metal waveguide together with the metallic ground plane. For one thing, the metamaterial layer will transfer the energy of the normal incident light into metamaterial resonant modes, which will be confined in the detector active region by the double-metal waveguide, as the same in Ref. 28. For another, the integrated device is expected to exhibit clearly polarization-dependent property due to the asymmetry of the metamaterial resonators. With normal incidence, significant enhancements of photocurrent response are obtained at the metamaterial resonances compared with the 45° edge facet coupling device. The photocurrent response enhancements exhibit clearly polarization dependence, and the largest response enhancement factor of 165% is gained for the incident light polarized parallel to the split-ring gap.

## Methods

**Fig. 1:**
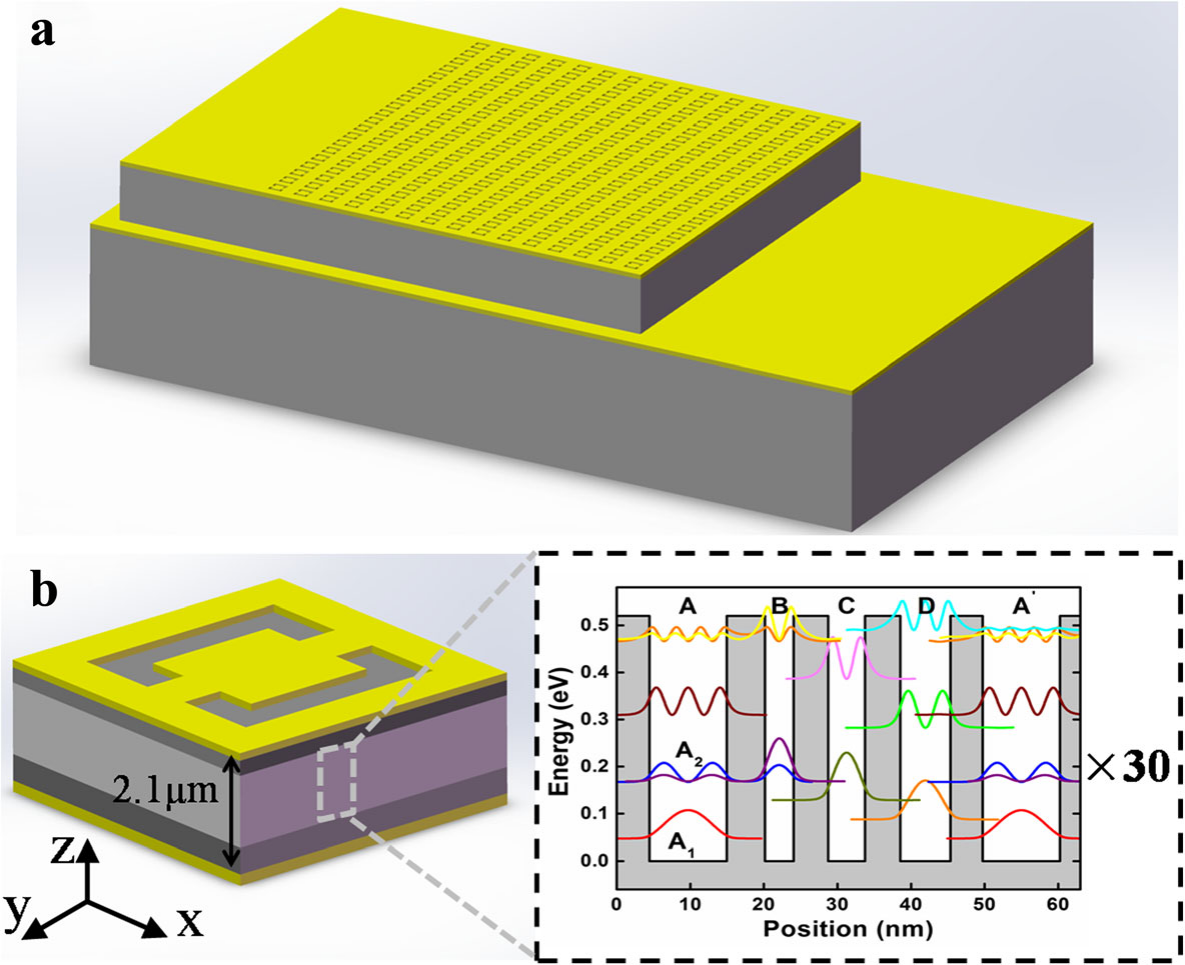
**a** Schematic of the metamaterial coupled detector. **b** Three-dimensional diagram for one metamaterial building block. Inset: self-consistently calculated conduction band structure of one period of the QCD. Starting with quantum well *A* from left to right, the layer thicknesses in angstroms are 105/52/39/47/50/48/68/44, where In_0.53_Ga_0.47_As wells are in *bold*, In_0.52_Al_0.48_As barriers are in *roman*, and the *underlined* active well *A* is Si-doped ($n$ = 4.5 × 10^17^ cm^−3^)

The quantum cascade detector used in this device was grown on a semi-insulating InP substrate by molecular beam epitaxy and designed to operate at a wavelength of 10.5 μm. The quantum cascade detector structure of one period is demonstrated in the inset of Fig. 1b in detail. The structure is a normal design, which is not well-designed for coupling with the metamaterial structure. The integrated device structure and the basic building block of the metamaterial are described schematically in Fig. 1. A 30-period chirped InGaAs/InAlAs superlattice (SL) active region and two Si-doped InGaAs(5 × 10^17^ cm^−3^) contact layers of 500 and 200 nm are sandwiched between an upper Au metamaterial layer and a bottom Au reflection layer whose thicknesses are both 100 nm. Thus, a double-metal waveguide structure is constituted to ensure a high overlap between the active detector region and the resonant mode excited by the metamaterial. Each building block of the metamaterial consists of a CSRR, which is directly fabricated into the top metal contact layer. Figure 1b also defines the coordinate system and shows the direction of the fundamental axes, which will be used throughout this work.

**Fig. 2:**
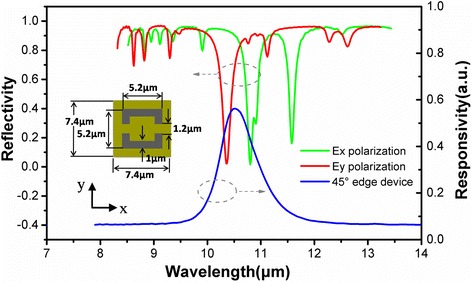
Simulated reflectance spectra for *E*_x_ and *E*_y_ polarization incident light, along with the detector’s origin response spectrum. Inset: the dimensions of the metamaterial structure obtained from FDTD simulation

**Fig. 3:**
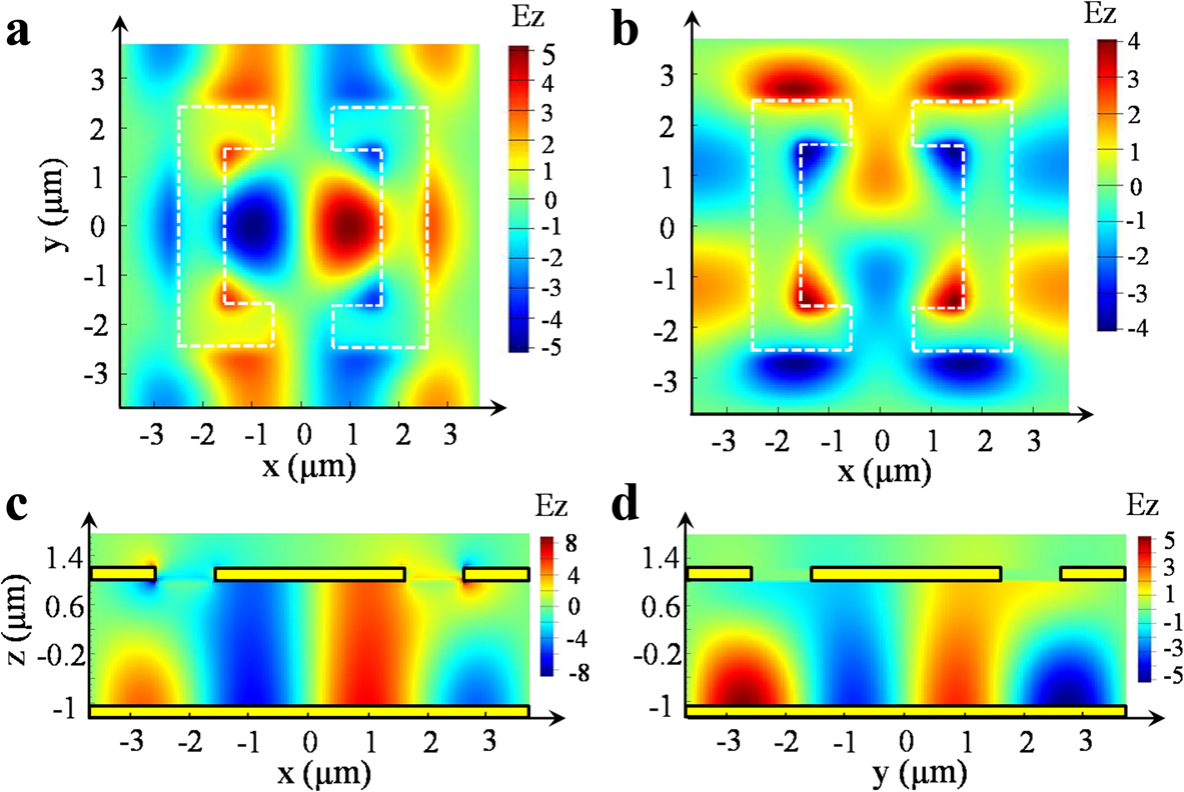
FDTD-simulated *E*_z_ electric field component distributions for the device. (**a**), (**b**) *E*_z_ electric component distribution in *x*–*y* plane 200 nm below the metamaterial layer for *E*_x_ polarization at 10.7 μm (panel (**a**)) and *E*_y_ polarization at 10.3 μm (panel (**b**)), respectively. (**c**), (**d**) *E*_z_ electric component distribution in *x–z* and *y–z* planes at *y* = 0 and *x* = 0 for *E*_x_ polarization at 10.7 μm (panel (**c**)) and for *E*_y_ polarization at 10.3 μm (panel (**d**)), respectively

The metamaterial properties, such as positions and line widths of the resonances, highly depend on the shape and size of the metallic resonators and can be fully controlled by the geometry of the resonant elements. In this work, higher order resonant modes of a complementary split-ring metamaterial are excited to interact with the intersubband transitions in the quantum cascade detector’s active origin. Three-dimensional FDTD simulations are performed for the structure design. Due to the Au film on the back, the structure does not allow light transmission, and therefore, its reflectance spectra is studied to characterize the metamaterial properties. The dips in the reflectance spectra correspond to the high metamaterial absorption and thus are the metamaterial resonances. According to the response spectrum obtained from the 45° edge facet coupling device, the dimensions of the metamaterial building block are determined and illustrated in the inset of Fig. 2. Due to the asymmetry in *x*–*y* plane, the CSRRs will have different resonate properties for the incoming light with different polarized directions. Figure 2 shows the simulated reflectance spectra for two polarization incident light, along with the response spectrum of the origin quantum cascade detector. Resonance wavelengths of 10.7 and 10.3 μm are gained for *E*_x_ and *E*_y_ polarization incident light, respectively, by geometric scaling of the CSRRs to overlap with the detector’s origin response spectrum with peak wavelength of 10.5 μm. In addition, there is another resonance at 11.5 μm on the edge of the response spectrum for *E*_x_ polarization, which will have similar coupled property as the resonance at 10.7 μm. To reveal the coupling mechanism in the integrated device, the *E*_z_ electric component distributions for the structure have been simulated and shown in Fig. 3. Figure 3a, b is *E*_z_ electric component distribution in *x*–*y* plane 200 nm below the metamaterial layer inside the semi-conductor for *E*_x_ polarization incident light at 10.7 μm and *E*_y_ polarization at 10.3 μm, respectively. The different spatial mode profiles for the *E*_x_ and *E*_y_ polarization incident light indicate that our device response goes beyond a simple grating coupler which would have the same response for both polarizations. Figure 3c, d is *E*_z_ electric component distributions in *x*–*y* and y–z planes at *y* = 0 and *x* = 0 for *E*_x_ polarization at 10.7 μm and for *E*_y_ polarization at 10.3 μm, respectively. They demonstrate the existence of strong *E*_z_ electric component through the whole active region confirming the significant confinements by the double-metal waveguide, which is vital for the device response enhancement.

**Fig. 4:**
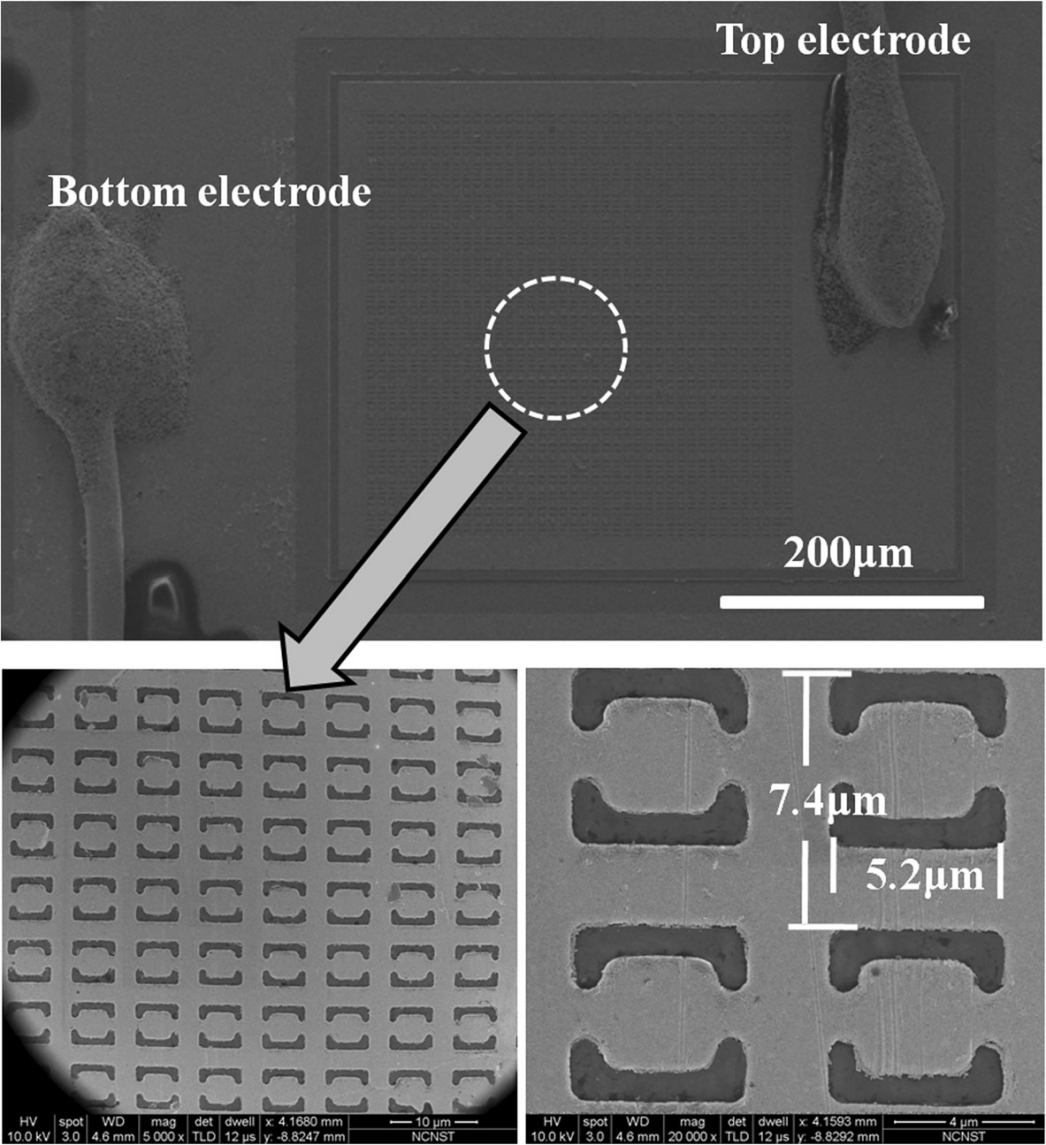
Scanning electron microscopy images of the integrated device (*top*) and CSRRs structure (*bottom*)

The device processing starts with the upside down mounting of the epitaxy wafer. After the deposition of a Ti(20 nm)/Au(100 nm) layer on the top contact layer, the epitaxial layer is fixed onto an InP receptor substrate using Deamcheas electricity conductive adhesive. Therefore, the Ti/Au layer deposited on the top contact layer will act as the reflection layer and bottom electrode in the integrated device. Following that, lapping and selective wet chemical etching using hydrochloric acid were performed to remove the original InP substrate and reach the InGaAs etch stop layer. Next, 300 × 400-μm^2^ device mesas are defined by standard photolithography and wet etching process. The mesa depth was controlled to reach the bottom reflection Au layer. Then 300 × 300-μm^2^ CSRRs metamaterial areas and 300 × 100-μm^2^ top electrodes were fabricated on the top of device mesas using a combination of standard photolithography and metal lift-off process. The CSRRs layer has a depth of 100 nm. The device fabrication was then completed with chip cleavage and gold wire bonding. In the top picture of Fig. 4, a large area scanning electron microscopy (SEM) image of the metamaterial integrated device is shown. The top and bottom electrical contacts are indicated in the figure. In the bottom picture of Fig. 4, the zoom-in views of the CSRRs are shown. The SEM images indicate that the morphology of CSRRs is not so idealized, which is limited to the method through which the CSRRs are obtained. However, this method is simple and of low cost and more promising for practical applications, such as fabricating large-area focal plane arrays.

## Results and Discussion

**Fig. 5:**
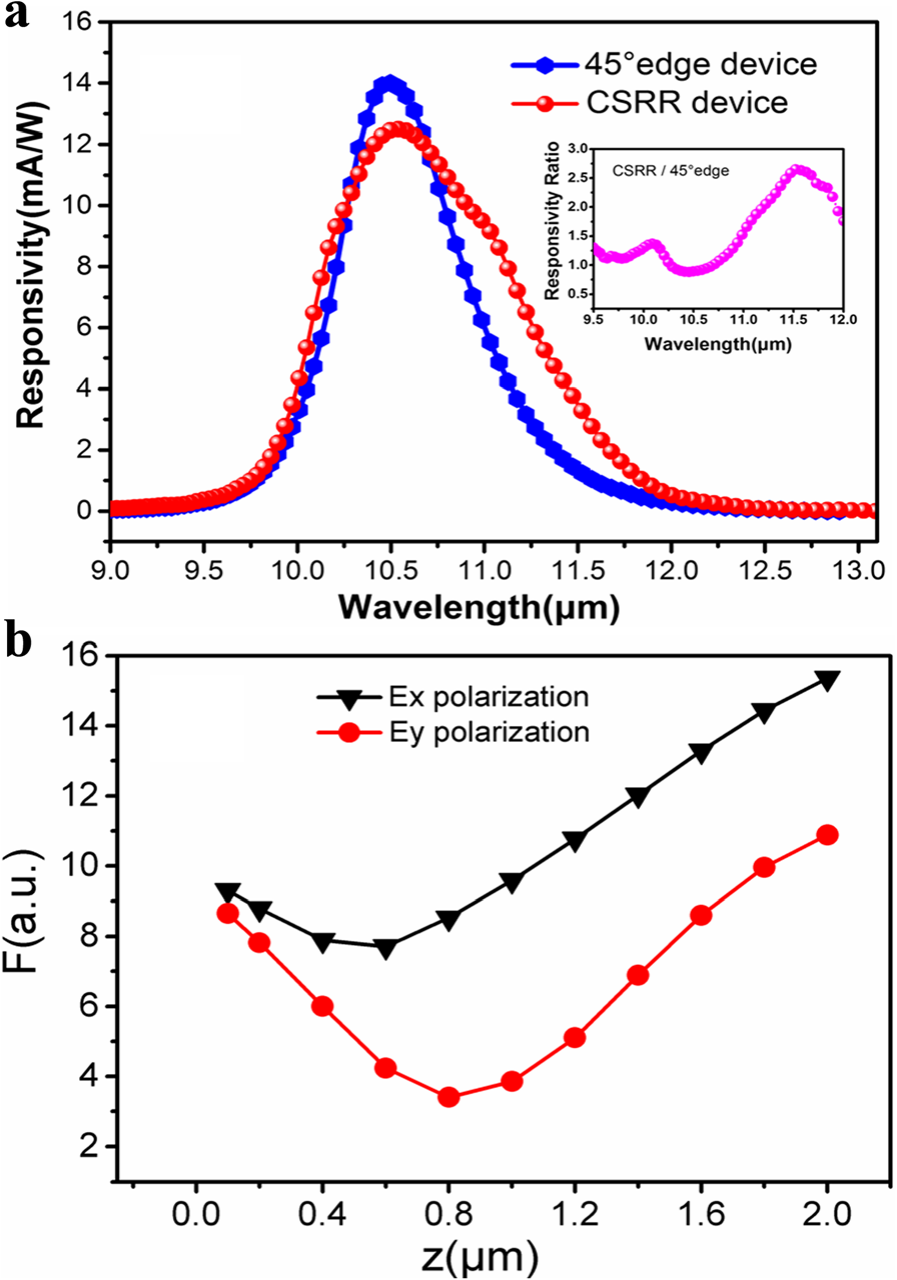
**a** Photocurrent responsivity of the CSRRs coupled device (*red*) together with that of 45° edge facet coupling device (*blue*). Inset: responsivity ratio of the CSRR device to 45° edge facet coupling device. **b** The z-dependences of the total amplitude for the *E*_x_ polarization at 10.7 μm (*black*) and *E*_y_ polarization at 10.3 μm (*red*)

Once the device processing is completed, it is mounted onto a copper heat-sink and then fixed on the cold finger of a liquid nitrogen cryostat for device response measurements. The photocurrent response measurement was performed by Nicolet 6700 fourier transform infrared spectrometer (FTIR) with a KBr beam splitter and the internal glow-bar illumination. With the measured photocurrent spectrum, the responsivity spectrums were calibrated using a standard blackbody source. Figure 5a shows the photocurrent responsivity spectra of the metamaterial integrated devices. Also shown is that of a 45° edge facet coupling device made from the same QCD wafer as the reference. In this integrated device, the expected detector response is a convolution of the intersubband transitions in detector’s active region and the metamaterial resonances. Therefore, the response spectrum shape of the CSRRs coupled device is clearly modified compared to the 45° edge facet coupling device. In addition, significant response enhancements are obtained at metamaterial resonances for both polarizations, as is shown in the inset of Fig. 5a. And the largest response enhancement factor of 165% is gained at the wavelength of 11.5 μm for the *E*_x_ polarization, which is far away from the peak response of the original device. This may be due to the low absorption for the original device, which will leave more electrons on the ground state for the metamaterial coupled absorption [30]. However, the response enhancement is not obtained in the whole response spectrum, which can be attributed to the narrow-banded property of the metamaterial resonances. In addition, at the peak response wavelength, incident light cannot coupled to the absorption region of the QCD structure efficiently through metamaterial resonances and thus the enhancement did not occur near the peak wavelength. Thus, in order to acquire enhancement through the whole response spectrum, metamaterial structures with broad-banded resonances should be designed and integrated to the detector [31]. Figure 5a also indicates that *E*_x_ polarization have stronger enhancement than the *E*_y_ polarization. To reveal the physical reason, the *z*-dependences of the total |*E*_*z*_| amplitude are calculated, which is obtained by taking the areal integral $F=\left|{\displaystyle {\int}_0^d{\displaystyle {\int}_0^d\left|{E}_z\left(x,y\right)\right| dxdy}}\right|$, for both *E*_x_ and *E*_y_ polarization incident light at metamaterial resonances of 10.7 and 10.3 μm, respectively, and is demonstrated in Fig. 5b. The results clearly demonstrated that the |*E*_*z*_| areal integrals for *E*_x_ polarization incident light are larger than those for *E*_y_ polarization at different *z* positions. This indicates that *E*_x_ polarization incident light can excite *E*_z_ electric field more efficiently and thus bring in stronger response enhancement. Moreover, there are clear dips in *F* curves at the middle of the waveguide. Since the coupled modes are excited by the metamaterial layer and confined by the double-metal waveguide, it is expected that the *E*_z_-field is strongest just underneath the top metal and close to the bottom metal contact. Due to the serial structure of QCD active region, the device responsivity will be limited by the region with small |*E*_*z*_| areal integral and thus low absorption. Therefore, further responsivity enhancement is in prospect by optimizing the position of absorption region in the waveguide, where the absorption region is layered at the positions with strongest *E*_z_ electric field. Another aspect that has to be considered is the ohmic losses due to the double-metal structure in the device, which will absolutely reduce the coupled energy in the detector and thus limit the response enhancement. In order to obtain exciting responsivity enhancement through metamaterial couple, special designed structures with low ohmic losses need to be investigated and employed in further study [32].

**Fig. 6:**
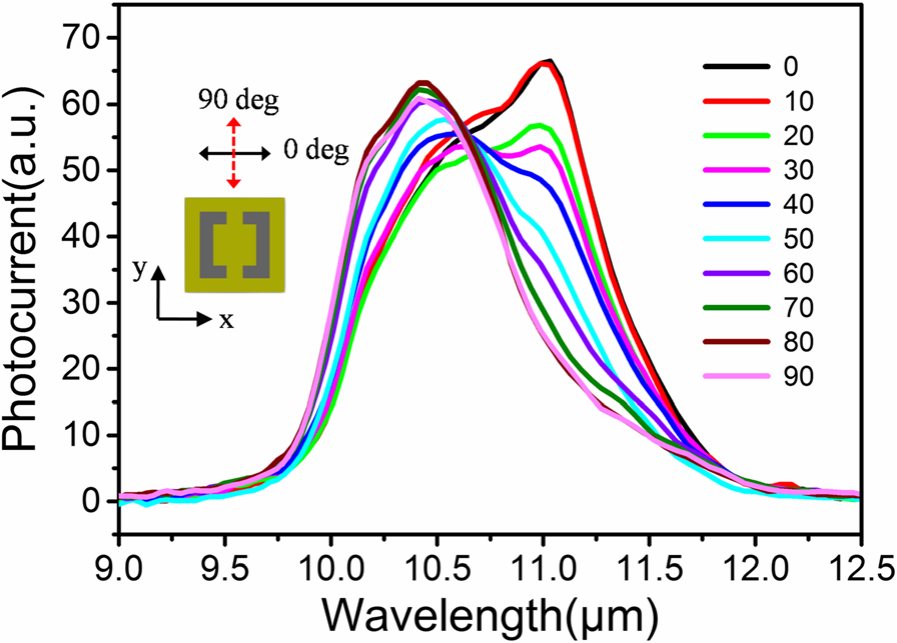
The photocurrent spectra at different polarization angles

To further gain the polarization-dependent property of the device, device photocurrent has been measured with different polarization incident light. Figure 6 is the photocurrent curves changing with the azimuth positions of the polarizer. 0° polarization corresponds to polarization in *x* direction and 90° in *y* direction, as is shown in the inset in Fig. 6. The results unambiguously show that the spectral responses are highly dependent on the incidence light polarization, which indicates the feasibility to design polarization-selective detectors using metamaterial resonances. However, the *E*_x_/*E*_y_ response ratio for this device is somewhat small. This can be due to the complex mutual effect of grating and metamaterial resonances, which is expected as the periodicity of the metamaterial is comparable to the effective wavelength inside the active region. In order to obtain high polarization discriminating detectors for practical application, such as polarimetric imaging, optimized metamaterial design should be implemented [33]. Furthermore, taking advantage of the unique properties of metamaterial, various metamaterial designs can be employed to implement multi-functional detector. For example, metamaterial structures with chirality can be applied to fabricate circular polarization discriminating detectors [34].

## Conclusions

In conclusion, a polarization-dependent normal incident quantum cascade detector coupled via complementary split-ring metamaterial resonators has been demonstrated. The metamaterial structure is fabricated directly into the top metal contact and forms a double-metal waveguide together with the metallic ground plane. With normal incidence, significant enhancements of photocurrent response are obtained at the metamaterial resonances compared with the 45° edge facet coupling device. And the largest response enhancement factor of 165% is gained at the wavelength of 11.5 μm for the *E*_x_ polarization. The response spectral also exhibits clearly polarization dependence, which outlines the advantages of metamaterial coupled design for polarization discriminating application. In further, taking advantage of the unique properties of metamaterial, many new IR detector functionalities can be implemented by designing suitable metamaterial structures, which presents a promising way for fabrication of high performance or special functional device for practical applications.

## Abbreviations

CSRR: Complementary split-ring resonator
FDTD: Three-dimensional finite-difference time-domain
FTIR: Fourier transform infrared spectrometer
QCDs: Quantum cascade detectors
SEM: Scanning electron microscopy
SL: Superlattice
